# What is the remaining status of adaptive servo-ventilation? The results of a real-life multicenter study (OTRLASV-study): Adaptive servo-ventilation in real-life conditions

**DOI:** 10.1186/s12931-019-1221-9

**Published:** 2019-10-29

**Authors:** Dany Jaffuel, Carole Philippe, Claudio Rabec, Jean-Pierre Mallet, Marjolaine Georges, Stefania Redolfi, Alain Palot, Carey M. Suehs, Erika Nogue, Nicolas Molinari, Arnaud Bourdin

**Affiliations:** 1 Department of Respiratory Diseases, Montpellier University Hospital, Hôpital Arnaud de Villeneuve, 371 avenue du Doyen Gaston Giraud, 34295 Montpellier, Cedex 5 France; 2 Pulmonary Disorders and Respiratory Sleep Disorders Unit, Polyclinic Saint-Privat, 34760 Boujan sur Libron, France; 3 Centre des pathologies du sommeil, Hôpital Universitaire de la Pitié Salpêtrière, AP-HP, Paris, France; 4 Pulmonary Department and Respiratory Critical Care Unit, University Hospital Dijon, Dijon, France; 5 Clinique des Bronches, Allergies et du Sommeil, Assistance Publique Hôpitaux de Marseille, Marseille, France; 6 INSERM U1067, CNRS UMR 7333 Aix Marseille Université, 13015 Marseille, France; 7 Hôpital Saint-Joseph, 26, boulevard de Louvain, 13285 Marseille, France; 8 Department of Medical Information, Montpellier University Hospital, Montpellier, France; 9 Clinical Research and Epidemiology Unit (URCE), Montpellier University Hospital, Montpellier, France; 10 IMAG, CNRS, Montpellier University, Montpellier University Hospital, Montpellier, France; 11 PhyMedExp (INSERM U 1046, CNRS UMR9214), Montpellier University, Montpellier, France

**Keywords:** Adaptive servo-ventilation, Central sleep apnea, Chronic heart failure, CPAP, Obstructive sleep apnea, Treatment emergent central sleep apnea, Sleep-disordered breathing

## Abstract

**Backgrounds:**

As a consequence of the increased mortality observed in the SERVE-HF study, many questions concerning the safety and rational use of ASV in other indications emerged. The aim of this study was to describe the clinical characteristics of ASV-treated patients in real-life conditions.

**Methods:**

The OTRLASV-study is a prospective, 5-centre study including patients who underwent ASV-treatment for at least 1 year. Patients were consecutively included in the study during the annual visit imposed for ASV-reimbursement renewal.

**Results:**

177/214 patients were analysed (87.57% male) with a median (IQ_25–75_) age of 71 (65–77) years, an ASV-treatment duration of 2.88 (1.76–4.96) years, an ASV-usage of 6.52 (5.13–7.65) hours/day, and 54.8% were previously treated via continuous positive airway pressure (CPAP). The median Epworth Scale Score decreased from 10 (6–13.5) to 6 (3–9) (*p* < 0.001) with ASV-therapy, the apnea-hypopnea-index decreased from 50 (38–62)/h to a residual device index of 1.9 (0.7–3.8)/h (*p* < 0.001). The majority of patients were classified in a Central-Sleep-Apnea group (CSA; 59.3%), whereas the remaining are divided into an Obstructive-Sleep-Apnea group (OSA; 20.3%) and a Treatment-Emergent-Central-Sleep-Apnea group (TECSA; 20.3%). The Left Ventricular Ejection Fraction (LVEF) was > 45% in 92.7% of patients. Associated comorbidities/etiologies were cardiac in nature for 75.7% of patients (neurological for 12.4%, renal for 4.5%, opioid-treatment for 3.4%). 9.6% had idiopathic central-sleep-apnea. 6.2% of the patients were hospitalized the year preceding the study for cardiological reasons. In the 6 months preceding inclusion, night monitoring (i.e. polygraphy or oximetry during ASV usage) was performed in 34.4% of patients, 25.9% of whom required a subsequent setting change. According to multivariable, logistic regression, the variables that were independently associated with poor adherence (ASV-usage ≤4 h in duration) were TECSA group versus CSA group (*p* = 0.010), a higher Epworth score (*p* = 0.019) and lack of a night monitoring in the last 6 months (*p* < 0.05).

**Conclusions:**

In real-life conditions, ASV-treatment is often associated with high cardiac comorbidities and high compliance. Future research should assess how regular night monitoring may optimize devices settings and patient management.

**Trial registration:**

The OTRLASV study is registered on ClinicalTrials.gov (Identifier: NCT02429986) on 1 April 2015.

## Introduction

Adaptive Servo-Ventilation (ASV) is a partially automated treatment modality used to correct various types of sleep-disordered breathing (SDB), including periodic breathing [1, 2], but also central and obstructive apnea and hypopnea [3–5]. Current proposed indications for ASV are Treatment-Emergent Central Sleep Apnea (TECSA), Central Sleep Apnea (CSA) associated with stroke, renal failure or other etiologies such as drug induced CSA, co-existing CSA with obstructive sleep apnea (OSA), and idiopathic CSA [5]. For patients with preserved LVEF (left ventricular ejection fraction, i.e. LVEF > 45%) and moderate-to-severe predominant CSA, ASV is an “*Option level recommendation*” according to the American Academy of Sleep Medicine (AASM) [6], whereas the European Respiratory Society Task Force proposed ASV in this clinical situation (but only after a Continuous Positive Airway Pressure (CPAP) trial failure) [5]. Based on the results of the SERVE-HF study [7], current recommendations underline a consensus against the use of ASV in Chronic Heart Failure (CHF) patients with both reduced LVEF (i.e. LVEF ≤45%) and moderate-to-severe predominant CSA [5, 6].

ASV was initially developed for the treatment of central sleep apnea and Cheyne-Stokes breathing associated with CHF and reduced LVEF [2]. Studies dedicated to these patients are somewhat relatively numerous as compared with other potential indications for ASV [4, 5, 8, 9] and in particular, the only large randomized study in the ASV field concerns these patients (SERVE-HF study, [7]). Paradoxicaly, the prevalence of related comorbidities/etiologies and sleep apnea patterns for real-life ASV populations has rarely been evaluated [8, 10–13]. Recently, in an unselected monocentric study concerning 293 ASV-treated patients, Randerath et al. reported that only 9.6% of the patients fulfilled the SERVE-HF criteria subtype, thus bringing into question the representativity of the patients included in previously published ASV-studies [13]. As a consequence of the increased mortality observed in the SERVE-HF study, many questions concerning the safety and rational use of ASV in other indications emerged [14].

With the aim of filling the literature gap characterized by a lack of studies describing associated comorbidities/etiologies for all types of ASV patients, we report here the clinical characteristics of the patients included in the Observational Transversal Real-life Study of ASV (OTRLASV) study. OTRLASV is a multicentric study aimed at describing the clinical characteristics of patients who have undergone ASV for over a year in real-life conditions.

## Methods

### Study design and study population

The OTRLSAV study is an observational prospective five-expert-centre study (see Additional file 1 for centres) conducted in a exhaustive cohort of consecutive patients treated for at least 1 year with ASV for sleep apnea (SA) (ClinicalTrials.gov Identifier: NCT02429986). The protocol complied with the Declaration of Helsinki and was reviewed and approved by an independent ethics committee (*Comité de Protection des Personnes “Sud Méditérannée III”*; reference number 2014.11.04).

SA was defined according to the French Social Security rules required for the reimbursement of ASV costs: 1) an Apnea Hypopnea Index (AHI) ≥ 30/h (or AHI ≥ 15/h and more than 10/h respiratory-effort-related arousal), and 2) associated with sleepiness and at least three symptoms from among snoring, headaches, hypertension, reduced vigilance, libido disorders, nycturia). In order to be reimbursed, the ASV-treated patient needs to be examined each year. Participating investigators enroled eligible patients (see Additional file 2 for inclusions/exclusion criteria) during this annual visit. Each investigative center was open for 14 months, starting in March 2015. The safety annoucement for the SERVE-HF study happened on May 13th, 2015. Prior to this, we included 8 patients (4.5%), and the remaing 169 patients (95.5%) were included after this date, with a last inclusion in January 2017).

### Collected data

The clinical information collected for the analysis included age, sex, anthropometry, smoking status, blood pressure, initial polysomnography (PSG) or respiratory polygraphy (PG) AHI, Epworth Sleepiness Scale (ESS), number of hospitalizations during the last year (with aetiology), presence of cardiomyopathy (with aetiology and treatment), especially an altered LVEF, cardiological monitoring, and whether or not the patient knew his/her drug prescription by rote.

The patient status for CHF and LVEF, neurological and renal comorbidities/etiologies, opioid prescriptions were systematically collected. An idiopathic CSA was defined when none of the above causes for CSA applied. ASV treatment modalities were also collected using the manufacturer’s software: usage reported in hours/night for the last 6 months, reported residual AHI by the device (AHI_flow_), auto-adjusted level of expiratory pressure use versus fixed expiratory pressure, inspiratory and expiratory pressure levels, duration of pressurization, backup frequency, leak level, interface type, use of humidifier, use of heated circuit, and use of a chinstrap. In addition, we collected the treatment initiation time and modality of initiation (hospital or ambulatory conditions), device and interface manufacturer, history of the devices used before ASV, history of the interfaces used. Whether or not night monitoring (a polygraphy / polysomnography / oximetry during ASV for 1 night) had been performed in the last 6 months was also collected, as well as any subsequent changes to device settings and interface choice.

### Initial polygraphy (PG) or polysomnography (PSG) diagnosis and definition of SDB groups

In line with a recent published real-life study [12], we chose to differentiate central versus obstructive groups using the predominant apnea pattern. Patients with more than 50% of central apneas were classified in the central sleep apnea (CSA) group, while patients with more than 50% of obstructives apneas were classified in the obstructive sleep apnea (OSA) group. Patients with an initial diagnosis of OSA treated with CPAP but secondarily treated with ASV were classified in the Treatment-Emergent Central Sleep Apnea (TECSA) group. Central apnea was scored if respiratory effort was absent. This latter criteria was chosen because it represented a consensus between the different centers and recommendations for scoring (see Additional file 3 for details).

### Echocardiography

All echocardiograms were performed by senior cardiologists. LVEF was calculated using the Simpson’s and/or Teichholz’s methods. For patients with multiple measures, only the most recent was kept for analysis, and a threshold of a LVEF ≤45% was used to categorize the patient as “reduced” versus “preserved” LVEF, as in the SERVE-HF study [7].

### Statistical analyses

Data distributions were assessed for normality and continuous data are expressed as means with their standard deviations (SD) or medians and interquartile ranges (IQ_25–75_) accordingly. Qualitative parameters were expressed as numbers and percentages. Comparisons between the three SDB-groups (CSA, OSA and TECSA) were performed using ANOVA or Kruskal-Wallis test for quantitative data. Qualitative variables were compared using Chi-square or Fisher test. For significant global comparison, pairwise comparisons were performed using Holm correction for multiple comparison.

The relationship between the date of ASV initiation and delays (since last echocardiography or the last echocardiography) was studied with the Cochran Armitage test. The relationship between the date of ASV initiation and a CPAP trial or a night monitoring in the 6 months preceding the inclusion of the patient in the study was studied using the Jonckheere-Terpstra test. ASV-usage groups were compared by Student’s test or Wilcoxon Mann Withney test for quantitative parameters and Chi-square or Fisher test for qualitative ones. A two-sided *p* value of < 0.05 was considered as indicating statistical significance.

Multivariable logistic regression analysis was used to study associations between ASV-adherence (≤4 h versus > 4 h) and collected data. Using backward selection, pertinent covariates with a univariable *p*-value < 0.15 were fed into the multivariable analysis. The α -to-exit was set at 0.05. Odds-ratios with their 95% Wald CI were reported. Model goodness-of-fit was assessed using the Hosmer-Lemeshow test. Missing data have not been replaced. All analyses were conducted by the Department of Research and Medical Information at the Montpellier University Hospitals using statistical software (SAS, V.9.3; SAS Institute; Cary, North Carolina, USA).

## Results

**Fig. 1:**
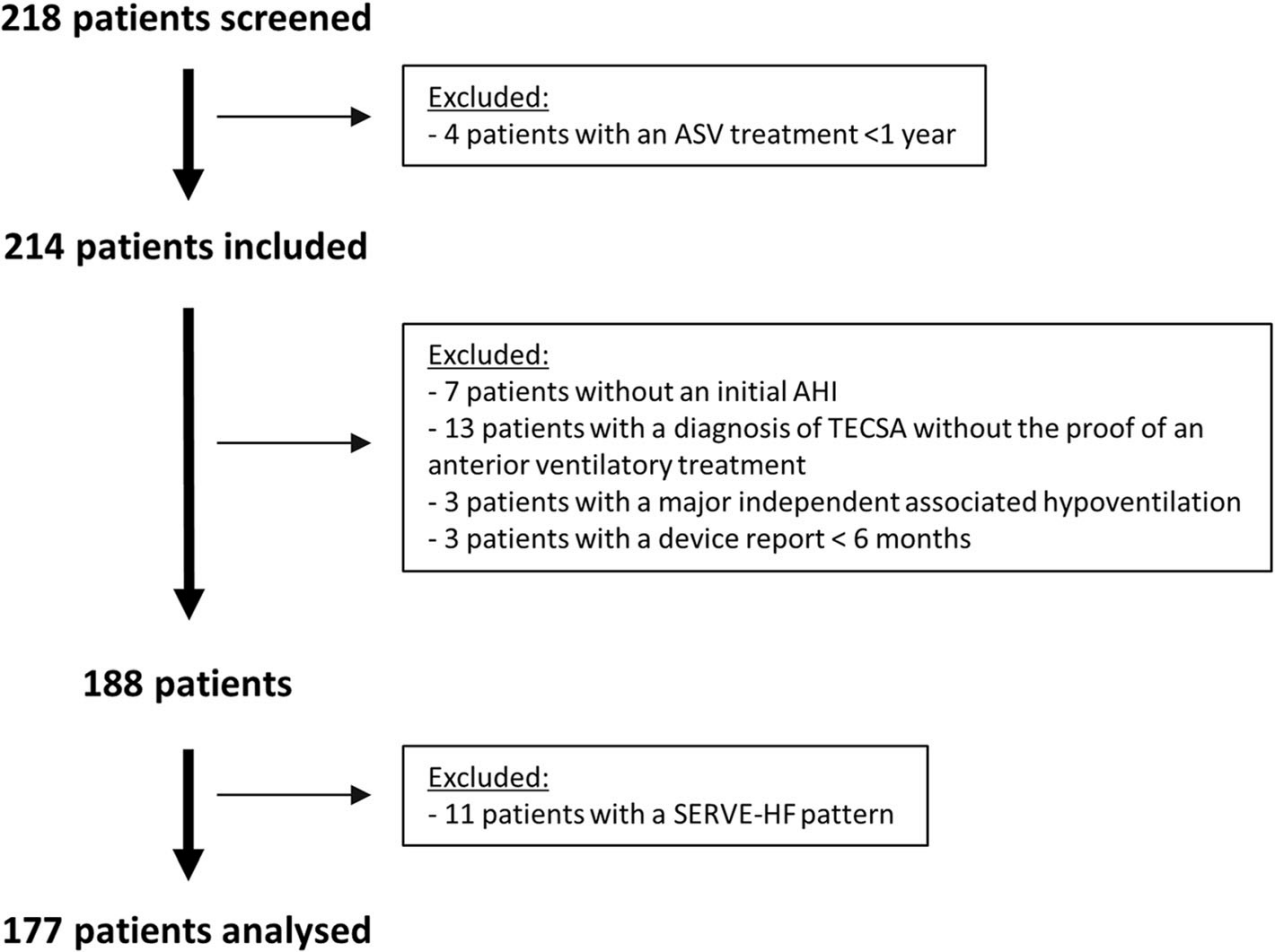
Flow chart of the study. ASV: Adaptive Servo-Ventilation; AHI: Apnea Hypopnea Index; TECSA: Treatment Emergent Central Sleep Apnea; SA: Sleep Apnea, SDB: Sleep Disordered Breathing

The flow chart for the study is depicted in Fig. 1. General and sleep characteristics of the population are summarised in Table 1. Briefly, the 177 patients (87.6% male) analysed had a median age of 71 (IQ_25–75_: 65–77) years, a median body mass index of 29.9 (26.6–34.0) kg/m^2^, and 12% were active smokers (35% had never smoked). The majority of patients was classified in the CSA group (59.3%), whereas the remaining 40.7% were evenly divided into an OSA group (20.3%) and a TECSA group (20.3%) (see Additional file 1 for SDB-group prevalence depending of the enrolment center). SDB-diagnosis was performed by PSG or PG in respectively 42.9 and 57.1% of cases. The median initial AHI for the whole population (WP) was 50/h (38–62), with no difference associated with the diagnosis method (AHI_PG_ of 50/h (39–57) versus AHI_PSG_ of 50/h (37–68), *p* = 0.729).

**Table 1:** General and sleep characteristics of the population

	N	Whole group, *n* = 177	CSA group, *n* = 105 (59.3%)	OSA group, *n* = 36 (20.3%)	TECSA group *n* = 36 (20.3%)	*P*
Anthropometric data						
Age (years)	177	71 [65–77]	71.00 [65.00–76.00]	69.50 [65.00–77.00]	74.50 [64.00–83.50]	0.447
Gender, n (%)	177					0.378
Male		155 (87.57%)	93 (88.57%)	33 (91.67%)	29 (80.56%)	
Female		22 (12.43%)	12 (11.43%)	3 (8.33%)	7 (19.44%)	
BMI (kg/m2)	175	29.90 [26.60–34.00]	29.80 [26.55–33.60]	29.10 [26.70–35.00]	31.55 [26.70–36.05]	0.530
Initial sleep data						
Initial exam						
PG		101 (57.06%)	55 (52.38%)	22 (61.11%)	24 (66.67%)	0.281
PSG		76 (42.92%)	50 (47.62%)	14 (38.89%)	12 (33.33%)	
Initial AHI (n/h)	177	50.00 [38.30–62.30]	50.00 [39.00–67.00]	46.80 [34.75–58.50]	47.05 [39.00–65.15]	0.671
Initial OAI (n/h)	154	7.70 [2.00–18.30]	4.00^ab^ [0.90–8.70]	16.45^b^ [9.80–21.80]	18.45^a^ [7.15–28.15]	<.001
Initial CAI (n/h)	154	10.75 [3.60–23.60]	17.00^ab^ [9.00–33.80]	7.50^bc^ [2.50–9.80]	3.50^ac^[0.65–7.70]	<.001
Initial MAI (n/h)	153	1.70[0.00–5.00]	1.50 [0.00–4.65]	4.00 [0.00–9.70]	0.75 [0.00–7.00]	0.279
Initial HI (n/h)	161	16.00 [8.70–24.90]	16.75 [8.70–24.30]	12.00 [8.35–23.50]	17.00 [11.00–27.00]	0.641
Initial ESS score	136	10.00 [6.00–13.50]	10.00 [6.00–13.00]	9.00 [4.00–14.00]	12.00 [6.50–13.50]	0.598
CPAP trial before ASV initiation, n (%)	166	91 (54.82%)	36^ab^ (37.11%)	19^bc^ (57.58%)	36^ac^ (100%)	< 0.001
Final sleep data						
Final AHI_flow_	177	1.90 [0.70–3.80]	1.80 [0.70–3.30]	1.95 [0.85–5.35]	2.25 [0.50–4.80]	0.448
Final ESS score	174	6.00 [3.0–9.0]	5.00 [3.0–9.0]	5.00 [2.0–10.0]	6.00 [3.0–10.0]	0.731
ASV-adherence						
Mean adherence > 4 h/day, n (%)	177	154 (87.01%)	99 (94.29%)^a^	30 (83.33%)	25 (69.44%)^a^	< 0.001

Quantitative variables were described by medians and [IQ_25_–_75_]. Significant pairwise comparisons after Holm correction were presented using ^a^ for CSA vs. ESA groups, ^b^ for CSA vs. OSA groups and ^c^ for OSA vs. ESA groups

*AHI* Apnea hypopnea index, *AHI*_*flow*_ Apnea Hypopnea Index estimated by the device, *BMI* Body mass index, *CAI* Central apnea index, *CPAP* Continuous positive airway pressure, *CSA* Central sleep apnea, *ESS* Epworth sleepiness scale, *HI* Hypopnea index, *MAI* Mixed apnea index, *OAI* Obstructive apnea index, *OSA* Obstructive sleep apnea, *PG* Polygraphy, *PSG* Polysomnography, *TECSA* Treatment emergent central sleep apnea

### ASV

A CPAP trial was performed before ASV initiation for 37.1% of the CSA group, 57.6% of the OSA group and 100% of the TECSA group (*p* < 0.001). The delay between the date of ASV initiation and the existence of a CPAP trial before ASV initiation is depicted in Additional file 4 (*p* = 0.37). No other mode of ventilation than ASV and CPAP was used.

For the WP, AHI indices significantly improved according to machine-derived values for the ASV treatment group (AHI_flow_ = 1.9/h (0.7–3.8)) versus pre-treatment PG/PSG-derived values (AHI_PG/PSG_ = 50.00 (38.30–62.30)], *p* < 0.001; Table 1). Significantly decreased final AHI_flow_ values were observed for each SDB group (versus initial AHI_PG/PSG_, *p* < 0.001). The median initial Epworth Scale Score (ESS) for the WP was 10 (6–13.5); the final ESS was 6 (3–9). The difference between initial and final ESS was significant for the CSA (*p* < 0.001) and TECSA groups (*p* = 0.009), but not for the OSA group (*p* = 0.068).

ASV initiation was performed at home for 35.3% of the WP, under hospital ambulatory conditions for 19.7 and 45.1% were admitted for continuous hospitalization (no differences were found between SDB groups, *p* = 0.162).

The median duration of ASV treatment was 2.88 years (1.76–4.96) with no difference between groups. The median ASV-usage for the WP was 6.5 h/day (5.1–7.7). 87.0% of the WP were adherent to ASV for more than 4 h/day. Table 2 depicts the comparison between sub-groups of ASV-adherence (≤4 h versus > 4 h) for clinical, ASV or monitoring data. Statistically significant differences existed (1) between SDB groups (*p* < 0.001), (2) for the presence of PG- or oximetry-based ASV monitoring in the last 6 months (*p* = 0.014), and (3) for the initial (*p* = 0.012) and final (*p* = 0.034) ESS scores.

**Table 2:** Comparison between ASV-adherence sub-groups (≤4 h versus > 4 h) for clinical, ASV and monitoring data

	N	≤4 h *N* = 23	> 4 h *N* = 154	*P*
Age (years)	177	74.00 [60.00;82.00]	71.00 [65.00;77.00]	0.964
Gender, n (%)	177			0.316
Female	22	1 (4.35%)	21 (13.64%)	
Male	155	22 (95.65%)	133 (86.36%)	
BMI (kg/m2)	175	29.40 [26.30;32.30)	30.10 [26.95;34.40]	0.379
SA sub-groups, n (%)	177			< 0.001
CSA	105	6 (26.09%)	99 (64.29%)	
OSA	36	6 (26.09%)	30 (19.48%)	
TESA	36	11 (47.83%)	25 (16.23%)	
Initial exam, n (%)	177			0.692
PG	101	14 (60.87%)	87 (56.49%)	
PSG	76	9 (39.13%)	67 (43.51%)	
Initial AHI (n/h)	177	50.00 [40.00;67.20]	50.00 [38.00;60.30]	0.636
Final AHI_flow_	177	2.00 [0.80;5.20]	1.85 [0.70;3.60]	0.362
Initial ESS score	136	12.50 [9.00;16.00]	9.00 [5.00;13.00]	0.012
Final ESS score	174	8.50 [4.00;12.00]	5.00 [3.00;9.00]	0.034
Initial ESS-final ESS score	136	2 (0.00–6.00)	2.50 (0.00–7.00)	0.775
ASV initiation during continuous hospitalization, n (%)	173	13 (61.90%)	65 (42.76%)	0.098
CPAP trial before ASV initiation, n (%)	166	16 (69.57%)	75 (52.45%)	0.126
Interface Type, n (%)	175			
Facial	87	12 (52.17%)	75 (49.34%)	0.800
Nasal/Nasal Pillows	88	11 (47.83%)	77 (50.66%)	
Cardiological comorbidity/etiology, n (%)	177	18 (78.26%)	116 (75.32%)	0.759
Neurological comorbidity/etiology, n (%)	177	0 (0.00%)	22 (14.29%)	0.053
Renal comorbidity/etiology, n (%)	177	2 (8.70%)	6 (3.90%)	0.278
Opiod comorbidity/etiology, n (%)	177	0 (0.00%)	6 (3.90)	0.336
Idiopathic CSA, n (%)	177	2 (8.70%)	15 (9.74%)	1.000
No comorbidity/etiology, n (%)	177	5 (21.74%)	28 (18.18%)	0.774
Patients with at least one hospitalization for cardiologic cause, n (%)	177	3 (13.04%)	8 (5.19%)	0.157
Number of cardiological medications	169	3.00 [1.00;4.00]	2.00 [1.00;3.00]	0.535
Knowledge of the medical treatment by the patient, n (%)	162	8 (40.00%)	83 (58.45%)	0.119
Echocardiography or cardiological consultation in the last 6 months, n (%)	144	11 (64.71%)	65 (51.18%)	0.294
Oxymetry or Polygraphy ASV control in the last 6 months	157	2 (10.00%)	52 (37.96%)	0.014
Modification of ASV settings as a consequence of Polygraphy or oximetry, n (%)	54	0 (0%)	14 (26.92%)	1.000

Quantitative variables were described by medians and [IQ_25_–_75_]

*AHI* Apnea hypopnea index, *AHI*_*flow*_ Apnea Hypopnea Index estimated by the device, *BMI* Body mass index, *CPAP* Continuous positive airway pressure, *CSA* Central sleep apnea, *ESS* Epworth sleepiness scale, *n* Number, *OSA* Obstructive sleep apnea, *PG* Polygraphy, *PSG* Polysomnography, *TECSA* Treatment emergent central sleep apnea, *SA* Sleep apnea

Multivariable logistic regression analysis was used to study associations between collected data and ASV-adherence (≤4 h versus > 4 h). The following variables (with a *p* < 0.15 value in the univariate analysis) were included in the multivariable model: SDB-groups, initial ESS, a PG- or oximetry-based ASV monitoring in the last 6 months, a CPAP trial before ASV initiation, an ASV initiation during continuous hospitalization, a neurological comorbidity, and patient treatment knowledge (whether or not the patient knew their treatment). Multivariable logistic regression demonstrated that (1) TECSA group versus CSA group, (2) absence of a PG- or oximetry-based ASV monitoring in the last 6 months and (3) a high initial EES score were associated with a ≤ 4 h ASV-adherence (Table 3). In order to rule out a possible confounding effect for the comorbidity variables, each “comorbidity/etiology” variable was forced into the multivariable analysis but the results were unchanged and “comorbidity/etiology” variables remained statistically non-significant.

**Table 3:** Logistic regression analysis with adherence (≤ 4 h /day) as the dependent variable. Summary of significant explicative variables

	Odds ratio [95% CI]	*P*-value
SA groups		*P* = 0.034
TECSA group versus CSA group	7.57 [1.063–35.21]	*p* = 0.010
OSA group versus CSA group	2.73 [0.49–15.27]	*p* = 0.252
Absence of night monitoring^a^ in the last 6 months	5.91 [1.003–34.82]	*p* = 0.0496
Initial EES score	1.18 [1.03–1.35]	*p* = 0.019

*CSA* Central sleep apnea, *ESS* Epworth sleepiness scale, *OSA* Obstructive sleep apnea, *PG* Polygraphy, *TECSA* Treatment emergent central sleep apnea. ^a^night monitoring: polygraphy- or oximetry-based ASV quality monitoring during an ASV night treatment in the last 6 months

### Comorbidities/etiologies reported for ASV-treated patients

**Fig. 2:**
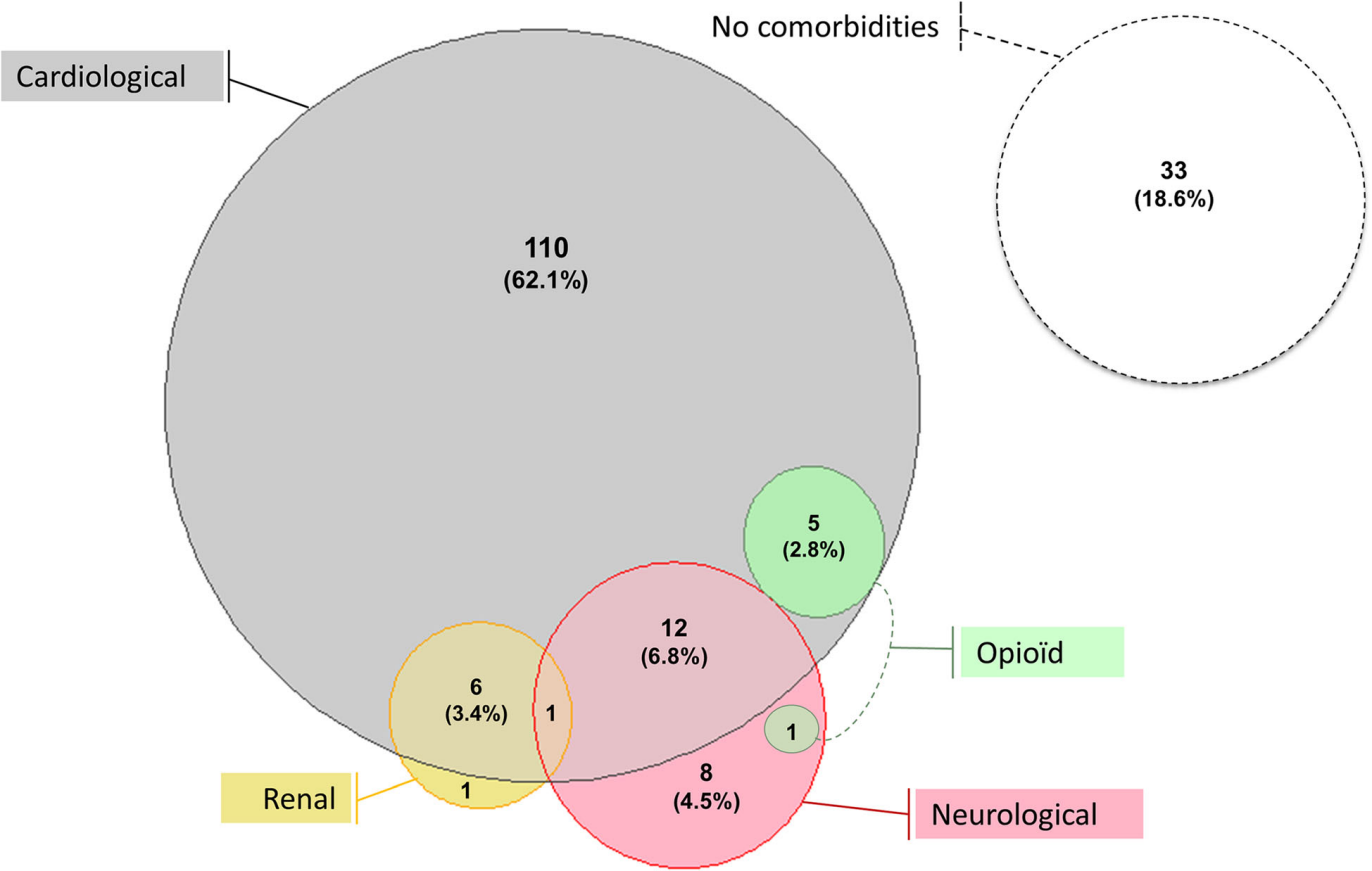
Comorbidities/etiologies associated with ASV prescription. The number and percentages of the total population are presented (1 patient = 0.6%). For 33 patients (18.6%), no comorbidity was related to SA

Associated comorbidities/etiologies are depicted in Fig. 2. Comorbidities/etiologies are strictly cardiological in nature for 62% of the patients, only neurological for 4%, and only renal failure for 0.5%. No patient had more than two comorbidities/etiologies and the vast majority (24/25) had at least one cardiological comorbidity. No comorbidities/etiologies were reported for 33 patients (18.6%), 51.5% of whom belonged to the CSA group. Thus, 9.6% of the WP can be defined as idiopathic CSA.

### Cardiological characteristic

Table 4 depicted the cardiological data of the population. Ischaemic heart failure was present in 34.9% of the WP, 24.6% presented with non-ischaemic heart failure, 30.5% with atrial fibrillation, and 7.3% were diagnosed with a reduced LVEF. For the 147 patients for whom the date of the last cardiological consultation was known, the median delay was 183 days (70–365). Similarly, the median delay since last echocardiography (*n* = 145) was 263 days (116–529) and appeared to differ between SDB groups (*p* = 0.015), with a shorter delay for the OSA group (175 days (28–356)). These delays were not dependent on the year of ASV initiation (Additional files 5 and 6, *p* = 0.19 and *p* = 0.77, respectively, for consultations and echocardiographic exams). 20.9% of patients were hospitalized the year preceding inclusion, but only 6.2% for cardiological reasons (in detail, six patients were hospitalized for acute (3) or chronic (3) coronary syndrome requiring revascularization by stent (5) or angioplasty (1), 3 patients for acute heart failure, 1 patient for acute atrioventricular block requiring implantation of a pacemaker and 1 patient for a stroke).

**Table 4:** Cardiovascular data

	N	Whole group, *N* = 177	CSA group, *N* = 105	OSA group, *N* = 36	TECSA group, *N* = 36	*P*
**Existence of cardiac disease, n (%)**	177	134 (75.71)	81 (77.14)	26 (72.22)	27 (75.00)	0.833
Ischaemic HF	175	61 (34.86%)	37 (35.58%)	11 (31.43%)	13 (36.11%)	0.891
Non Ischaemic HF	175	43 (24.57%)	26 (25.00%)	9 (25.71%)	8 (22.22%)	0.931
Valvulopathy	175	13 (7.43%)	4 (3.85%)^c^	6 (17.14%)^c^	3 (8.33%)	0.025
History of AF	174	53 (30.46%)	32 (31.07%)	11 (31.43%)	10 (27.78%)	0.925
Other cardiac disease	175	33 (18.86%)	18 (17.31%)	4 (11.43%)	11 (30.56%)	0.098
**Cardiological monitoring**						
Cardiological consultation, n (%)	151	147 (97.35%)	89 (95.70%)	26 (100.00%)	32 (100.00%)	0.467
Delay since the last consultation (days)^a^	147	183 [70–365]	188.0 [80.0–365]	117.5 [24–262]	214.5 [125–470]	0.070
Cardiological echocardiography, n (%)	148	145 (97.97%)	89 (97.80%)	25 (100.00%)	31 (96.88%)	1.000
Delay since the last echocardiography (days)^a^	145	263 [116–529]	266^c^ [113–541]	175^d^ [28–356]	315^cd^ [172–665]	0.015
**Hemodynamic parameters** ^a^						
Systolic BP (mmHg)	149	130 [118–140]	130.0 [119.0–140.0]	130.0 [111.0–40.00]	131.0 [114.0–147.0]	0.740
Diastolic BP (mmHg)	149	75 [70–82]	75.00 [70.00–80.00]	78.50 [66.00–85.00]	74.00 [70.00–85.00]	0.937
Heart Rhythm (bpm)	155	70 [62–77]	70.00 [62.00–76.00]	68.00 [60.00–78.00]	70.00 [63.00–77.00]	0.876
LVEF, n (%)	177					< 0.001
Reduced (LVEF ≤45%)		13 (7.34%)	0 (0.00%)^bc^	8 (22.22%)^c^	5 (13.89%)^b^	
Normal		164 (92.6%)	105 (100.00%)	28 (77.78%)	31 (86.11%)	
**Treatment, n (%)**						
Diuretic	168	73 (43.45%)	39 (37.14%)	18 (56.25%)	16 (51.61%)	0.097
Spironolactone	166	19 (11.45%)	12 (11.65%)	4 (12.50%)	3 (9.68%)	1.000
ACE inhibitor	168	61 (36.31%)	36 (34.29%)	13 (40.63%)	12 (38.71%)	0.771
β-receptor blocker	168	64 (38.10%)	38 (36.19%)	12 (37.50%)	14 (45.16%)	0.663
ARB	165	35 (21.21%)	24 (23.53%)	7 (21.88%)	4 (12.90%)	0.446
Calcium blocker	169	38 (22.49%)	23 (21.90%)	11 (33.33%)	4 (12.90%)	0.144
Cardiac glycoside	168	2 (1.19%)	2 (1.90%)	0 (0.00%)	0 (0.00%)	1.000
Antiarrhythmic drug	168	24 (14.29%)	12 (11.43%)	6 (18.75%)	6 (19.35%)	0.326
Antiagregants	168	45 (26.79%)	25 (23.81%)	8 (25.00%)	12 (38.71%)	0.250
Anticoagulant	168	37 (22.02%)	22 (20.95%)	8 (25.00%)	7 (22.58%)	0.887
Pacemaker	175	22 (12.57%)	12 (11.54%)	4 (11.43%)	6 (16.67%)	0.664
ICD	175	7 (4.00%)	0 (0%)^bc^	3 (8.57%)^c^	4 (11.11%)^b^	0.002
**Hospitalization during the preceding year**						
Patients with at least one hospitalization for any cause, n (%)	177	37 (20.90%)	19 (18.10%)	7 (19.44%)	11 (30.56%)	0.276
Patients with at least one hospitalization for a cardiologic cause, n (%)	177	11 (6.21%)	5 (4.76%)	3 (8.33%)	3 (8.33%)	0.509

*ACE* Angiotensin-converting enzyme, *AF* Atrial fibrillation, *ARB* Angiotensin-receptor blocker, *BP* Blood pressure, *CSA* Central sleep apnea, *HF* Heart failure, *ICD* Implanted cardiac defibrillator, *LVEF* Left ventricular ejection fraction, *OSA* Obstructive sleep apnea, *TECSA* Treatment emergent central sleep apnea

^a^Quantitative variables were described by medians and [IQ_25_–_75_]. Significant pairwise comparisons after Holm correction were presented using ^b^ for CSA vs. ESA groups, ^c^ for CSA vs. OSA groupas and ^d^ for OSA vs. ESA groups

### Polygraphy and oximetry-based ASV monitoring data

**Fig. 3:**
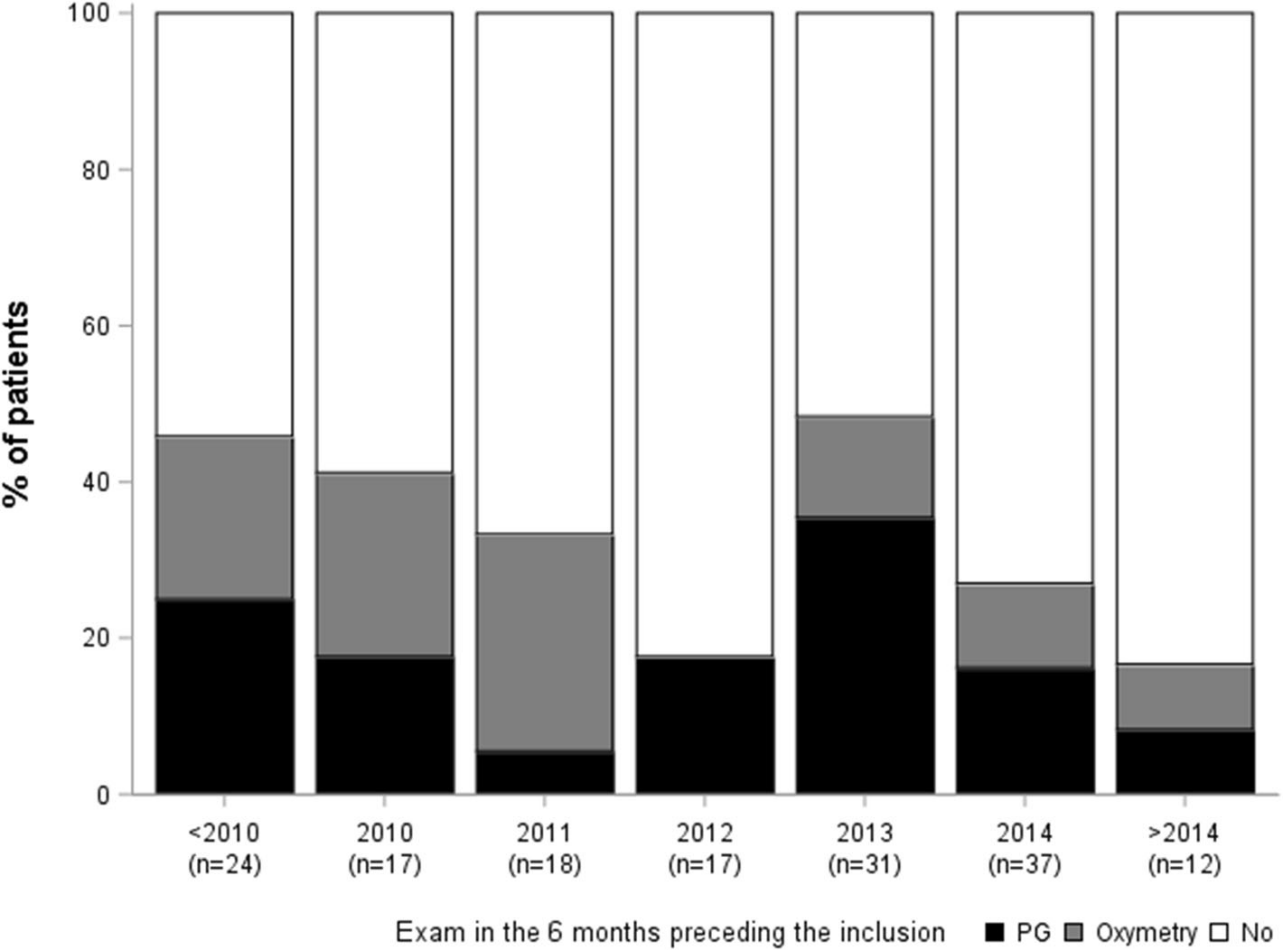
Exams (polygraphy (PG) or oximetry) performed in the 6 months preceding study inclusion, depending of the ASV-initiation date

Data for PG- or oximetry-based ASV monitoring performed in the 6 months preceding inclusion are summarised in Table 5. PG on ASV was performed in 31/173 patients, whereas 24/160 patients had overnight oximeter recording on ASV; one patient has both types of control. These controls were associated with a consecutive change in settings for 7 patients in either group (ASV-PG *n* = 7 and ASV-oximetry *n* = 7). These changes consisted in a modification of the pressure level for 9 patients, with a modification of the back-up frequency rate for one patient, and a modification of the interface for 5 patients. The cases where a PG- or oximetry-based ASV monitoring was performed in the last 6 months were not linked with the ASV-initiation date (*p* = 0.12, see Fig. 3).

**Table 5:** Data from polygraphy- or oximetry-based ASV quality monitoring performed in the last 6 months preceding the inclusion in the study

	N	Whole group *N* = 177	CSA group *N* = 105	OSA group *N* = 36	TECSA group *N* = 36	*P*
Polygraphy, n (%)	173	31 (17.9%)	18 (17.5%)	7 (20.6%)	6 (16.7%)	0.897
Apnea Hypopnea Index, (n/h)	31	1.90 [0.4;4.2]	1.50 [0.4;2.4]	3.5 [0.4;21.9]	1.55 [0.2;4.2]	0.578
Apnea Index, (n/h)	31	0.0 [0.0;0.2]	0.0 [0.0;0.2]	0.10 [0.00;2.70]	0.0 [0.0;0.2]	0.369
Hypopnea Index, (n/h)	31	1.9 [0.4;3.9]	1.3 [0.4;2.4]	3.5 [0.2;11.8]	1.5 [0.9;3.9]	0.659
ODI 3%, (n/h)	30	6.9 [3.9;11.6]	4.7 [2.4;7.2]	9.1 [7.5;23.9]	11.3 [4.5;19.9]	0.056
Mean SpO2, (%)	30	95.2 [94.0;96.0]	95.5 [94.8;96.0]	95.0 [93.0;95.9]	94.0 [92.70;96.0]	0.379
Modification of ASV settings as a consequence of polygraphy, n (%)	31	7 (22.6%)	2 (11.1%)	3 (42.9%)	2 (33.3%)	0.138
Oximetry, n (%)	160	24 (15.0%)	17 (17.9%)	4 (11.8%)	3 (9.7%)	0.531
ODI (n/h)	24	3.2 [1.5;9.7]	2.8 [1.9;10.9]	3.5 [2.1;5.8]	8.7 [1.1;10.7]	0.908
Mean SpO2, (%)	24	93.5 [92.0;94.0]	93.1 [91.4;94.0]	94.0 [93.0;96.0]	93.6 [93.0;93.6]	0.478
Modification of ASV settings as a consequence of oximetry, n (%)	24	7 (29.2%)	6 (35.3%)	0 (0%)	1 (33.33%)	0.519

Quantitative variables were described by medians and [IQ_25_–_75_]

*CSA* Central sleep apnea, *ESS* Epworth sleepiness scale, *ODI* Oxygen desaturation index, *OSA* Obstructive sleep apnea, *PG* Polygraphy, *TECSA* Treatment emergent central sleep apnea

## Discussion

In the context of the SERVE-HF study [7], a trial that has raised serious concerns about the effect and safety of ASV, physicians are waiting for new related studies [15]. Our study provides new data on ASV-use in real-life conditions and new insights for future trials. We report that: 1) the major comorbidity associated with ASV-treated patients after SERVE-HF study remains cardiologic in nature, and concerns 75.7% of patients (but, only 6.2% of the latter were hospitalized for cardiological reasons during the preceding year); 2) 54.8% of the ASV-treated patients previously received a CPAP treatment; 3) 87.0% of the patients were adherent to their ASV for more than 4 h/day; 4) more than a third of the patients included in our study had polygraphy- or oximetry-based monitoring to verify ASV quality in the 6 months preceding inclusion and a consecutive change (device settings or mask type) was performed for 25.9% of them. Interestingly, this monitoring was positively associated with an ASV-adherence > 4 h/jour.

### Conditions associated with ASV

This prospective, real-life study on a non-selected ASV population from five French centers is the first to give data on the related comorbidities/etiologies in “post-SERVE-HF” conditions (see Table 6 to compare with other, similar, real-life studies). In our study, the more prevalent associated comorbidities/etiologies were cardiac in nature for 75.7% of patients (59.5% of the WP present with CHF and 30.5% present with atrial fibrillation). In the Rochester Epidemiology Project (REP) database, a similar high prevalence for cardiac comorbidities/etiologies (78%) and atrial fibrillation (35.9%) was reported, but with less heart failure (34%) [14].

**Table 6:** Data from the published ASV-real-life and non comparative studies (only studies with more than 70 patients were included; data concern the whole population)

	N / C	Main sub-groups analysis reported	Prevalence of related SA comorbidity/etiology	CPAP trial before ASV	Duration of ASV / ASV-adherence	Initial AHI/h / Final AHI/h or AHI_*flow*_*/h*	Initial Epworth / Final Epworth
Carnevale et al., 2011 [10]. Retrospective	74 / 2	55% non-CHF and 45% CHF	NA CHF with LVEF≤45% NA CHF with LVEF> 45% 17% N, NA R, NA O, 28% I	15/74 patients Duration of the trial NA	36 ± 18 months / 75.6% > 3 h/jour	53.0 ± 23.8/h / 5.9 ± 8.0/h	8.9 ± 5.3 / NA
Momomura et al., 2015 [11]. Retrospective	115 / 16	24% ASV-discontinued CHF and 76% ASV-continued CHF	NA CHF (LVEF≤45%) NA CHF (LVEF> 45%) NA N, NA R, NA O, NA I	No CPAP trial	NA / NA	28.8 ± 19.2/h for ASV-discontinued CHF and 24 ± 21.3/h for ASV-continued CHF/NA	NA / NA
Malfertheiner et al., 2017 [12]. Retrospective	285 / 2	32% Cardiac center 68% Pulmonary center	39% CHF with LVEF≤45% 40% CHF with LVEF> 45% 0% N, NA R, 0.4% O, 10% ICSA	1 night for 86 CSA patients and median trial of 17 days for 178 OSA patients	NA / NA	NA / NA	9 ± 4.5 / NA
Randerath et al., 2017 [13]. Retrospective	293 / 1	57% CSA, 36% OSA, and presence of risk criteria (LVEF ≤45% and CSA)	16% CHF with LVEF≤45% 23% CHF with LVEF> 45% NA N, NA R, 8% O, NA I	NA	NA / NA	46.4 ± 20.5/h / NA	7.8 ± 4.5 / 5.4 ± 3.7
Oldenburg et al., 2019 [8]. Retrospective	224 / 1	100% CHF and LVEF ≤45% and AHI ≥ 15/h with predominant central pattern	NA	No CPAP trial	24 months 65.9% > 4 h/day at 24 months	37.7 ± 13.4 / 2.8 ± 3.2/h at 24 months	NA / NA
Jaffuel et al. Prospective	177 / 5	59.3% CSA, 20.3% OSA, 20.3% TECSA (11 patients with LVEF≤45% and CSA were excluded)	7.3% CHF with LVEF≤45% 51.4% CHF with LVEF> 45% 12.4% N, 4.5% R, 3.4% O, 9.6% I	91/177 Duration of the trial NA	34.5 (21.1–59.5) months / 87% > 4 h/day	50/h (38–62) / 1.9/h (0.7–3.8)	10 (6–13.5) / 6 (3–9)

*AHI* Apnea hypopnea index, *AHI*_*flow*_ Apnea hypopnea index estimated by the device, *CHF* Chronic heart failure, *CSA* Central sleep apnea, *TECSA* Treatment emergent central sleep apnea, *I* Idiopathic CSA, *LVEF* Left ventricular ejection fraction, *N/C* Number of patients and centres, *N* Neurological comorbidity/etiology, *NA* Not available, *O* Opioid comorbidity/etiology, *OSA* Obstructive sleep apnea, *R* Renal comorbidity/etiology, *SA* Sleep apnea. Results are expressed as means ± SD or medians and quartiles as reported in the original publication

To date, the prevalence of idiopathic CSA is unknown [5]. The 9.6% prevalence of idiopathic CSA found in our study is close to the 10% reported by the recent study from Malfertheiner et al. [12], but differs from the 28% given by the only previous report in 2011 [10]. In the REP database, the prevalence of idiopathic CSA was only 4.9% [14]. It is impossible to determine if these differences between studies are the consequence of a recruitment bias related to the investigative centers, the absence of collected data or a real change in the prevalence of the comorbidities/etiologies associated with the prescription of ASV. In particular, the prevalence of idiopathic CSA is conditioned by the exhaustively aetiologic screening performed, which is not always specified in real-life studies (e.g. cerebral screening with magnetic resonance imaging). Surprisingly, there are no recent recommendations concerning the aetiological screening to be carried out as a prerequisite for ASV prescription, except for a cardiac evaluation to rule out the possibility of a reduced LVEF in CSA patients [5].

### CPAP trials as a prerequisite for ASV therapy

For patients with CSA and failure of a recommended first-line CPAP trial, the 2017 European Respiratory Society Task Force systematically proposed ASV therapy as a second line of therapy (except for SERVE-HF pattern patients for whom ASV is contraindicated) [5]. The same recommendation exists for OSA patients [5] (and is a defining characteristic of TECSA patients). In contrast, in 2012, CPAP treatment for CSA patients was only an “Option level” recommendation for the American Academy of Sleep Medicine [7].

Here, we report that only 37.1% of the patients in the CSA group and 57.6% of the OSA group had a CPAP trial prior to ASV therapy. The percentage of CPAP trials occurring before ASV intiation remains stable over time, and therefore appears to not be influenced by the different recommendations. In other, similar, published real-life studies (Table 6), the required pre-ASV CPAP trial was not always performed, and when performed, lacked important specifications and/or appropriate duration.

In a recent, large, manufacturer-maintained database, it was surprising to observe that only 3.6% of the 9295 patients treated with ASV were previously treated with CPAP, thus questioning the true prevalence of TECSA-patients treated with ASV [16]. However, this type of manufacturer-database cannot rule out the possibility of a previous CPAP treatment with a different manufacturer, and thus underestimating the TECSA-prevalence. In contrast, the prevalence of TECSA was 75.5% of the ASV-treated patients in the REP database [14]. The exact role CPAP screening among patients eligible for ASV treatment should be detailed in future studies.

### ASV-adherence

One of the major criticisms of the SERVE-HF study was the weak ASV-adherence of the patients. Indeed, only 47% of the patients were adherent for more than 4 h/day at 1 year (with a mean of only 3.4 h/day). The data presented for the CAT-HF study were even worse, with 2.7/h/day at 6 months [17]). In contrast, 87% of our patients were adherent for more than 4 h/day. This high adherence was also reported by the French study from Carnevale et al. [10], and is likely linked to the reimbursement rules imposed by the French single-payer national insurance system. Unfortunately, ASV-adherence or usage was rarely reported in the other, similar, real-life studies, except for the Oldenburg et al. study (65.9% of patients > 4 h/day at 24 months, see Table 6) [8]. A recent analysis of a large database from the United States confirms a 73.2% ASV-adherence at 3 months for 8957 patients without previous CPAP trials in real-life conditions (ASV-adherence defined by an ASV usage ≥4 h per night, > 70% of nights during the consecutive 30-day period preceding the collection of the data). In the same study, the ASV-adherence at 3 months was 76% for the 209 patients who were previously CPAP treated [16], which is similar to the 69.4% reported in our study.

However, to date, an ASV-usage dependent effect on quality of life has not been demonstrated, as was the case for CPAP [11, 18, 19]. In the CAT-HF trial, the relationship between ASV-adherence (> 3 h) and the burden associated with atrial fibrillation does not reach significance despite a beneficial effect of combined optimal medical treatment (OMT) plus ASV-treatment versus OMT alone [20].

ASV-adherence is of crucial meaning because it is difficult to imagine a potential effect of ASV on strong outcomes (such as quality of life or cardiovascular mobility or mortality) without greater adherence than those reported in the recent ASV-trials [7, 17]. Of course ASV-adherence is a complex parameter, underlined by the on-treatment analysis of the SERVE-HF study. Indeed, Woehrle et al. reported that patients randomised to control who voluntarily switched to ASV had lower cardiovascular mortality than those initially randomised to ASV [21]. In addition, if the increase in cardiovascular mortality is associated with ASV, the risk did not appear to be proportional to the duration of ASV-use [21]. ASV-adherence may not be only a marker of ASV-therapy, but also a marker of a wide-range of patient behaviours toward health and disease. In this regard, it was suggested that ASV usage may be linked to oral medication compliance [22]. For CPAP therapy and OSA patients, two previous studies have reported conflicting results [23, 24]. In our study, we failed to demonstrate a link between the ASV-adherence and the number of cardiological medications or patient knowledge concerning his/her drug treatments. In the REP database, the adherence to ASV at any time was not associated with the rate of change of medication pre-ASV versus post-ASV [14]. Future ASV-randomized studies should assess oral medication compliance in order take to rule out possible bias when interpreting ASV effects [22]. This is one of the major criticisms against the SERVE-HF design study [22].

### Polygraphy and oximetry-based ASV monitoring

One of the interesting insights from our study concerns the PG- and oximetry-based ASV quality monitoring and the subsequent consequences on settings and ASV-adherence. 34.4% of patients were so monitored, and a consecutive setting change was then performed among 25.9% of them. ASV quality monitoring was not linked to the ASV initiation date, but was favourably associated with ASV-adherence. During ASV therapy, few studies report the correlation and concordance of the AHI measured by PG or PSG and the simultaneous AHI results given by the ASV device (AHI_flow_) (i.e. real versus device-provided measures). For CPAP, it was underlined that AHI_flow_ was not always correlated or concordant with PG/PSG measures, especially when a 3% versus a 4% threshold of oxygen desaturation is used (results were worse when a PSG was used because of the additive impact of arousals (which cannot be diagnosed by the device) on the scoring) [18, 25–27]. Equivalent, exhaustive data are lacking for ASV therapy, whereas preliminary [28] or final data [8, 29, 30] are in favour of a similar discrepancy between AHI_flow_ and AHI_PSG_. In the Silveira study, the Bland and Altman plot of the difference between PSG-AHI and ASV-AHI_flow_ against the mean of both measurements, reports a mean difference of 11.9 ± 9.6 (95% limits of agreement − 6.90, 30.71) [30]. In a recent editorial, Thomas and Bianchi have underlined the existing concern that the efficacy of CPAP and ASV therapies can be overestimated by the reported AHI_flow_ [27]. Future randomized ASV-studies must take into account these considerations by including several PSG controls for ASV quality in the study design. The latter should rule out the consequences of non-optimised ASV therapy on mechanistic parameters such as arousal and desaturation, which are innately underestimated by ASV AHI_flow_. This is of crucial importance considering the potential ineffectiveness of the device suggested by the literature and the possible consequences on ASV-adherence suggested by our study.

### Limits of the study

Our prospective study is a non-randomized observational study with potential unknown sources of bias. Large randomized controlled studies are needed, but a preliminary step is a careful assessment of patients currently treated or potentially eligible for ASV treatment. Observational studies must be multicenter to eliminate bias related to patient recruitment (cf. Additional file 2).

In constrast with recent, similar, real-life studies, our study was not specifically designed to assess the prevalence of SERVE-HF pattern patients in the ASV-treated population. Prevalences of 9 and 12% for SERVE-HF pattern patients were respectively reported in retrospective studies by Randerath et al. [13] and Malfertheiner et al. [12], whereas we report only a 5.8% prevalence. The chronology of our study and the release-date for the SERVE-HF safety notice explains this apparent discrepancy. Our first inclusion occurred in March 2015; the safety notice was released in May 2015. Therefore, our prospective study probably underestimated the prevalence of these patients, because most of the patients stopped their ASV treatment after the safety notice (in this regard, no SERVE-HF pattern patients were included in the 3 centers that joined the study after October 2015). An additional limitation of our study arises from one of the inclusion criteria. Indeed, we were unable to collect the occurrence of spontaneous improvement in central sleep apnea because only patients presenting at the annual control consultation for the continuation of the ASV treatment were included in the study.

Of course, our data may be less relevant to other countries mainly because of governmental policy rules governing ASV-costs. In France, ASV reimbursement at the time of this study was based on a combination of associated clinical symptoms, an AHI-threshold (regardless of apnea and hypopnea patterns) and an ASV-usage > 3 h/day. As a consequence, patients with a diagnostic AHI < 15/h were not treated with ASV, unlike patients included in other real-life studies [11, 12].

The major problem we faced was to classify patients into the CSA and OSA groups according to the results of their PV or PSG exams. As in Malfertheiner et al. [12], we chose to differentiate central versus obstructive SDB groups using the predominant apnea pattern. This choice helped overcome problems caused by changes in scoring recommendations for respiratory events. Indeed, in our study, patient initial diagnoses spanned from 2002 to 2016. During this period, the definition of apnea remained stable, whereas the definition of hypopnea went through major changes, including not only decreased thresholds for the percentage of flow, but also 3% or 4% oxygen desaturation thresholds, and central versus obstructive pattern definitions [31, 32].

In contrast to the consequences of not performing PG- or oximetry-based ASV-night monitoring, we failed to report the consequences of the cardiologic consultation and echocardiography (in particular in terms of cardiological therapy or ASV-setting changes). Future trials must record these data because modifications in the cardiologic treatment can bias the evaluation of ASV-therapy.

## Conclusion

Real-life studies inherently have many biases, but they can help us to better construct randomized studies. Our study reports the updated prevalence of cardiological, neurological, renal and opioid comorbidities/etiologies associated with ASV prescriptions. It emphasizes the need to better define CPAP as a prerequisite for ASV, and emphasizes the need for iterative night-monitoring and cardiological assessments in ASV-treated patients.

## Supplementary information

**Additional file 1.** SDB patient groups and enrolment center.

**Additional file 2.** Inclusion and exclusion criteria.

**Additional file 3.** Definition of the Central Sleep Apnea Group, the Obstructive Sleep Apnea group, and the Treatment Emergent Central Sleep Apnea Group.

**Additional file 4. **Relationship between the existence of a CPAP trial before ASV initiation and the date of ASV initiation (*p* = 0.37).

**Additional file 5. **Date of the last cardiological consultation depending on the year of ASV initiation (*p* = 0.19).

**Additional file 6. **Date of the last cardiological echocardiography depending on the year of ASV initiation (*p* = 0.77).

## Data Availability

The datasets used and/or analyzed during the current study are available from the corresponding author on reasonable request.

## Abbreviations

AASM: American Academy of Sleep Medicine
ACE: Angiotensin-converting enzyme
AF: Atrial fibrillation
AHI: Apnea Hypopnea Index
AHI_flow_: Residual Apnea–Hypopnea-Index measured by the ASV device
APH Marseille: Assistance Publique Hopitaux de Marseille
APHP Paris: Assistance Publique Hopitaux de Paris
ARB: Angiotensin-receptor blocker
ASV: Adaptive Servo-Ventilation
BMI: Body mass index
BP: Blood pressure
CAI: Central Apnea Index
CHF: Chronic Heart Failure
CHU Dijon: Centre Hospitalier Universitaire de Dijon
CHU Montpellier: Centre Hospitalier Universitaire de Montpellier (CHU Montpellier)
CPAP: Continuous Positive Airway Pressure
CSA: Central Sleep Apnea
ESS: Epworth Sleepiness Scale
HI: Hypopnea Index
ICD: Implanted cardiac defibrillator
IQ_25–75_: Medians and interquartile ranges
LVEF: Left ventricular ejection fraction
MAI: Mixed Apnea Index
OAI: Obstructive Apnea Index
OSA: Obstructive sleep apnea
PC Boujan: Polyclinique Saint Privat Boujan sur Libron
PG: Respiratory polygraphy
PSG: Polysomnography
SA: Sleep Apnea
SD: Standard deviations
SDB: Sleep-disordered breathing
TECSA: Treatment Emergent Central Sleep Apnea
WP: Whole population
