# Supplementary material for: What is the remaining status of adaptive servo-ventilation? The results of a real-life multicenter study (OTRLASV-study): Adaptive servo-ventilation in real-life conditions
Source: Respir Res. 2019 Oct 29;20:235. doi: 10.1186/s12931-019-1221-9 (PMC6819598; doi:10.1186/s12931-019-1221-9)
Supplement: Supplementary file 1 — Additional file 1. SDB patient groups and enrolment center. [file 12931_2019_1221_MOESM1_ESM.docx]

Additional file 1.

**SDB patient groups and enrolment center.**

|  | **Whole group**  **n=177** | **CSA group,**  **n=105** | **OSA group,**  **n=36** | **TECSA group**  **n=36** |
| --- | --- | --- | --- | --- |
| **Center** n (%) |  |  |  |  |
| APH Marseille | 27 (15.25%) | 26 (24.76%) | 0 (0%) | 1 (2.78%) |
| APH Paris | 42 (23.73%) | 18 (17.14%) | 16 (44.44%) | 8 (22.22%) |
| CHU DIJON | 45 (25.42%) | 33 (31.43%) | 5 (13.89%) | 7 (19.44%) |
| CHU Montpellier | 29 (16.38%) | 7 (6.67%) | 7 (19.44%) | 15 (41.67%) |
| PC Boujan | 34 (19.21%) | 21 (20.00%) | 8 (22.22%) | 5 (13.89%) |

APH Marseille: Assistance Publique Hopitaux de Marseille; APHP Paris : Assistance Publique Hopitaux de Paris; CHU Dijon : Centre Hospitalier Universitaire de Dijon; CHU Montpellier: Centre Hospitalier Universitaire de Montpellier (CHU Montpellier); CSA: Central Sleep Apnea; OSA: Obstructive Sleep Apnea; PC Boujan : Polyclinique Saint Privat Boujan sur Libron; TECSA: Treatment Emergent Central Sleep Apnea, SDB: Sleep-Disordered Breathing.
