# Supplementary material for: What is the remaining status of adaptive servo-ventilation? The results of a real-life multicenter study (OTRLASV-study): Adaptive servo-ventilation in real-life conditions
Source: Respir Res. 2019 Oct 29;20:235. doi: 10.1186/s12931-019-1221-9 (PMC6819598; doi:10.1186/s12931-019-1221-9)
Supplement: Supplementary file 2 — Additional file 2. Inclusion and exclusion criteria. [file 12931_2019_1221_MOESM2_ESM.docx]

Additional file 2.

**Inclusion and exclusion criteria.**

| Inclusion criteria | Exclusion criteria |
| --- | --- |
| - 18 years of age or older - Patient treated with ASV for at least one year, and eligible for care and for the reimbursement by the French Social Security rules - Patient presenting at the annual control consultation for the continuation of the ASV treatment according to the French Social Security rules - Available ASV-report device for the 6 months preceding the consultation. - Signed informed consent | - Pregnancy - Inability to understand the nature and aims of the study or to communicate with the investigator - Simultaneous participation in another trial with an exclusion clause to participate in another trial - No affiliation with the French social security regime - Loss of personal capacity resulting in state protection - Weinmann® device - Deprivation of liberty by judicial or administrative decision |

ASV: Adaptive servoventilation
