# Supplementary material for: What is the remaining status of adaptive servo-ventilation? The results of a real-life multicenter study (OTRLASV-study): Adaptive servo-ventilation in real-life conditions
Source: Respir Res. 2019 Oct 29;20:235. doi: 10.1186/s12931-019-1221-9 (PMC6819598; doi:10.1186/s12931-019-1221-9)
Supplement: Supplementary file 3 — Additional file 3. Definition of the Central Sleep Apnea Group, the Obstructive Sleep Apnea group, and the Treatment Emergent Central Sleep Apnea Group. [file 12931_2019_1221_MOESM3_ESM.docx]

Additional file 3

**Definition of the Central Sleep Apnea Group, the Obstructive Sleep Apnea group, and the Treatment Emergent Central Sleep Apnea Group.**


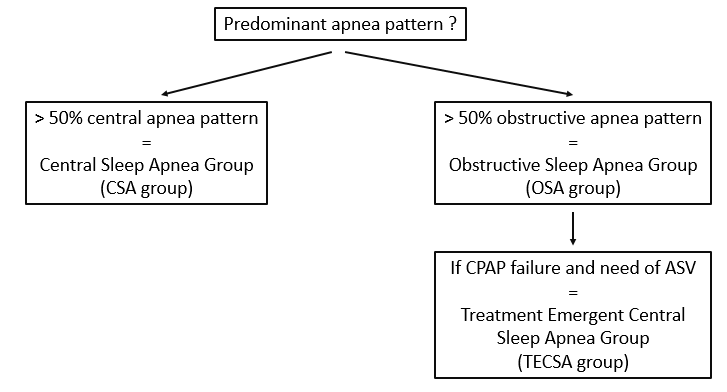


Diagnostic methods (polygraphy (PG) or polysomnography (PSG) ), and the scoring of respiratory events were not standardised (excepted for central or obstructive apnea patterns) because of the change overtime in recommendations for the scoring of respiratory events (initial PG or PSG diagnoses span 2002 to 2016).
