# Supplementary material for: What is the remaining status of adaptive servo-ventilation? The results of a real-life multicenter study (OTRLASV-study): Adaptive servo-ventilation in real-life conditions
Source: Respir Res. 2019 Oct 29;20:235. doi: 10.1186/s12931-019-1221-9 (PMC6819598; doi:10.1186/s12931-019-1221-9)

Additional file 4.

**Relationship between the existence of a CPAP trial before ASV initiation and the date of ASV initiation (p=0.37).**


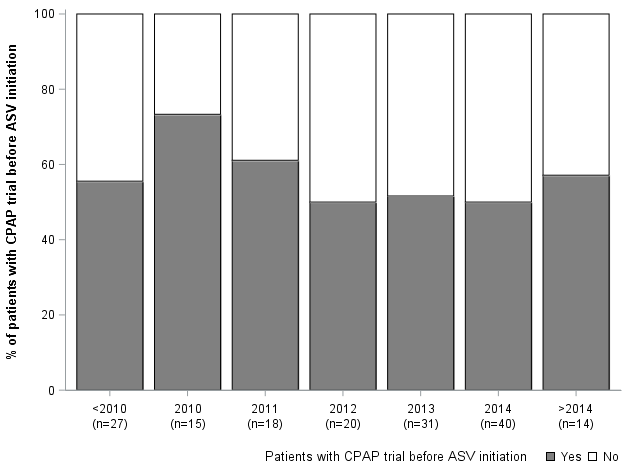

Supplement: Supplementary file 4 — Additional file 4. Relationship between the existence of a CPAP trial before ASV initiation and the date of ASV initiation (p = 0.37). [file 12931_2019_1221_MOESM4_ESM.docx]
