# Supplementary material for: What is the remaining status of adaptive servo-ventilation? The results of a real-life multicenter study (OTRLASV-study): Adaptive servo-ventilation in real-life conditions
Source: Respir Res. 2019 Oct 29;20:235. doi: 10.1186/s12931-019-1221-9 (PMC6819598; doi:10.1186/s12931-019-1221-9)
Supplement: Supplementary file 5 — Additional file 5. Date of the last cardiological consultation depending on the year of ASV initiation (p = 0.19). [file 12931_2019_1221_MOESM5_ESM.docx]

Additional file 5.

**Date of the last cardiological consultation depending on the year of ASV initiation (p=0.19 )**


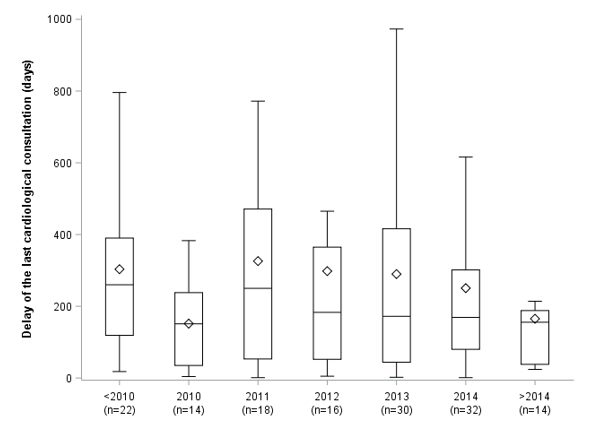


Bold line is median, square is mean, box is first quartile to the third quartile (IQR). Whiskers are minimum and maximum (or Q1- 1.5 IQR and Q3+ 1.5 IQR respectively ; values above 1.5*IQR from the box are considered to be outliers and not represented)
